# Lsr2 Is an Important Determinant of Intracellular Growth and Virulence in *Mycobacterium abscessus*

**DOI:** 10.3389/fmicb.2019.00905

**Published:** 2019-04-30

**Authors:** Vincent Le Moigne, Audrey Bernut, Mélanie Cortès, Albertus Viljoen, Christian Dupont, Alexandre Pawlik, Jean-Louis Gaillard, Fabienne Misguich, Frédéric Crémazy, Laurent Kremer, Jean-Louis Herrmann

**Affiliations:** ^1^2I, UVSQ, INSERM, Université Paris-Saclay, Versailles, France; ^2^UMR 9004, Centre National de la Recherche Scientifique, Institut de Recherche en Infectiologie de Montpellier, Université de Montpellier, Montpellier, France; ^3^VitamFero, Tours, France; ^4^Unité de Pathogénomique Mycobactérienne, Institut Pasteur, Paris, France; ^5^APHP, GHU PIFO, Hôpital Raymond-Poincaré – Hôpital Ambroise-Paré, Boulogne-Billancourt, France; ^6^INSERM, Institut de Recherche en Infectiologie de Montpellier, Montpellier, France

**Keywords:** non-tuberculous mycobacteria, *Mycobacterium abscessus*, Lsr2, virulence, pathogenesis, zebrafish, mouse

## Abstract

*Mycobacterium abscessus*, a pathogen responsible for severe lung infections in cystic fibrosis patients, exhibits either smooth (S) or rough (R) morphotypes. The S-to-R transition correlates with inhibition of the synthesis and/or transport of glycopeptidolipids (GPLs) and is associated with an increase of pathogenicity in animal and human hosts. Lsr2 is a small nucleoid-associated protein highly conserved in mycobacteria, including *M. abscessus*, and is a functional homolog of the heat-stable nucleoid-structuring protein (H-NS). It is essential in *Mycobacterium tuberculosis* but not in the non-pathogenic model organism *Mycobacterium smegmatis*. It acts as a master transcriptional regulator of multiple genes involved in virulence and immunogenicity through binding to AT-rich genomic regions. Previous transcriptomic studies, confirmed here by quantitative PCR, showed increased expression of *lsr2* (*MAB_0545*) in R morphotypes when compared to their S counterparts, suggesting a possible role of this protein in the virulence of the R form. This was addressed by generating *lsr2* knock-out mutants in both S (Δ*lsr2*-S) and R (Δ*lsr2*-R) variants, demonstrating that this gene is dispensable for *M. abscessus* growth. We show that the wild-type S variant, Δ*lsr2*-S and Δ*lsr2*-R strains were more sensitive to H_2_O_2_ as compared to the wild-type R variant of *M. abscessus*. Importantly, virulence of the Lsr2 mutants was considerably diminished in cellular models (macrophage and amoeba) as well as in infected animals (mouse and zebrafish). Collectively, these results emphasize the importance of Lsr2 in *M. abscessus* virulence.

## Introduction

*Mycobacterium abscessus* is a rapidly growing mycobacterium (RGM) increasingly acknowledged as a serious non-tuberculous mycobacterial (NTM) pathogen (Mougari et al., 2016; Diel et al., 2017). Although it causes extrapulmonary infections (Jeong et al., 2017) and disseminated pulmonary diseases among otherwise healthy individuals (Varghese et al., 2012), it has become notorious for the serious threat it poses to patients with cystic fibrosis (CF) and with other underlying lung disorders (Brown-Elliott and Wallace, 2002; Medjahed et al., 2010). *M. abscessus* infection correlates with a decline in pulmonary function and is an appreciable concern for CF patients requiring lung transplantation (Esther et al., 2010; Qvist et al., 2016; Smibert et al., 2016). *M. abscessus* has high intrinsic levels of resistance to many antibiotics, making infections with this mycobacterium hard to treat and eradicate (Brown-Elliott and Wallace, 2002; van Dorn, 2017).

*Mycobacterium abscessus* presents either smooth (S) or rough (R) colony morphotypes leading to different clinical outcomes (Howard et al., 2006; Catherinot et al., 2009; Medjahed and Reyrat, 2009; Ripoll et al., 2009; Roux et al., 2011). They also exhibit different morphological aspects (Howard et al., 2006; Sánchez-Chardi et al., 2011) and virulence phenotypes (Byrd and Lyons, 1999; Catherinot et al., 2007; Bernut et al., 2014a). These differences rely mainly on the presence (in S) or absence (in R) of surface-associated glycopeptidolipids (GPL) (Gutiérrez et al., 2018). The S variant is thought to be the colonizing form, capable of producing mature biofilms and exhibiting a significant motility on soft agar (Howard et al., 2006). The R variant, on the other hand, is impaired in these abilities and forms pronounced serpentine cords, a feature associated with hypervirulence (Byrd and Lyons, 1999; Catherinot et al., 2007; Bernut et al., 2014a; Halloum et al., 2016). Being the most pathogenic RGM, it is not surprising that both variants of *M. abscessus* resist phagocytosis by immune cells, a trait shared with pathogenic slow-growing mycobacterial (SGM) species, such as *M. tuberculosis* (Oberley-Deegan et al., 2010; Nessar et al., 2011; Roux et al., 2016). Therefore, the S-to-R transition, which occurs essentially *in vivo* and is responsible for a significant increase in pathogenicity (Jönsson et al., 2007; Rottman et al., 2007; Bernut et al., 2014b) likely represents an evolutionary adaptation mechanism to the host immune response (Pawlik et al., 2013; Roux et al., 2016).

Genome comparison of three distinct isogenic S/R couples of *M. abscessus* revealed the presence of genetic lesions in the R variant, such as single nucleotide polymorphisms and/or insertions/deletions, in genes belonging to the GPL biosynthesis and transport locus (Pawlik et al., 2013) and causing the S-to-R transition. Although these mutations can account for the change in colony morphology of *M. abscessus*, it is unlikely that they fully explain the virulence of the R variant observed in multiple cellular and animal models (Catherinot et al., 2007; Bernut et al., 2014b, 2017). This view is supported by transcriptomic studies performed in the same isogenic S/R pairs, which revealed differential expression for a large set of genes, including *lsr2* (Pawlik et al., 2013).

Lsr2 is a nucleoid-associated protein (NAP) and a functional analog of the heat-stable nucleoid-structuring proteins or H-NS (Chen et al., 2008; Gordon et al., 2008; Qu et al., 2013) that is conserved in all actinomycetes and mycobacteria (Chen et al., 2008; Gordon et al., 2008; Qu et al., 2013). Lsr2 was originally shown to be an immunodominant antigen of *Mycobacterium leprae* (Laal et al., 1991) and has since been reported to regulate a broad range of processes (Chen et al., 2008; Colangeli et al., 2009; Gordon et al., 2010). In *Mycobacterium smegmatis*, *lsr2* mutants show a different colony morphology and are defective in biofilm formation, a phenotype presumably resulting from an altered expression of key surface lipids, such as mycolyl-diacylglycerols (Chen et al., 2006; Arora et al., 2008; Kocíncová et al., 2008; Yang et al., 2017). Subsequent studies also demonstrated that Lsr2 is necessary for mycobacterial conjugal DNA transfer (Nguyen et al., 2010). In contrast to *M. smegmatis*, in which it is dispensable for planktonic growth, Lsr2 is considered essential in *M. tuberculosis* (Chen et al., 2006; Colangeli et al., 2007; Arora et al., 2008; Kocíncová et al., 2008; Yang et al., 2017). In addition, it has also been shown to contribute to antibiotic resistance (Colangeli et al., 2007) and to control the expression of a large panel of genes acquired by horizontal gene transfer in *M. tuberculosis* (Gordon et al., 2010). Like H-NS, Lsr2 preferentially binds to AT-rich sequences and can oligomerize through its protein-protein interaction domain to form filaments along the *M. tuberculosis* chromosome (Gordon et al., 2010, 2011). Moreover, it has the ability to bridge distant DNA segments, suggesting a role in the organization and compaction of the nucleoid (Chen et al., 2008).

Herein, we generated *lsr2* knock-out mutants in both S and R variants of *M. abscessus* to define the contribution of Lsr2 in major physiological processes that are relevant to the context of increased pathogenicity of the R variant.

## Materials and Methods

### Strains and Culture Media

*Mycobacterium abscessus* subsp. *abscessus* S and R 19977-IP strains were used in this study and designated Mabs-S and Mabs-R, respectively. Mycobacterial strains were grown aerobically at 37°C in Middlebrook 7H9 broth or on Middlebrook 7H11 agar, supplemented with 0.2% glycerol and 1% glucose. When necessary; kanamycin, hygromycin, and zeocin were added to medium at 250 μg/ml, 500 μg/ml (or 1000 μg/ml for selection) and 25 μg/ml, respectively. After infection experiments, bacterial CFU were counted on agar plates (BioMérieux, France), either Columbia blood agar plates after infections in macrophages and amoeba, and H_2_O_2_ exposition, either VCAT (Vancomycin, Colistin sulfate, Amphotericin B, and Trimethoprim) chocolate agar plates after mice infections. The *Escherichia coli* TOP10 strain (Thermo Fisher) was grown in Lysogeny Broth (LB) medium with or without kanamycin (25 μg/ml).

### Construction of *lsr2* Mutants in *M. abscessus*

The *lsr2* downstream and upstream regions were amplified by PCR using the primer pairs MC75/MC76 and MC80/MC81, respectively, then cloned with the zeocin resistance cassette yielding pMC34. Replacement of the endogenous *lsr2* gene by the zeocin resistance cassette was performed as described previously (van Kessel and Hatfull, 2007; Medjahed and Reyrat, 2009; Bakala N’Goma et al., 2015). *lsr2* knock-out mutants, designated Mabs-S-Δ*lsr2* and Mabs-R-Δ*lsr2* were confirmed by PCR (primer pairs lsr2-5/lsr2V and MC75/ZeoR) (Supplementary Figure S1) and Southern blotting (Figure 2B). Complementation was achieved by PCR amplification of *lsr2* under the control of its endogenous promoter region (961 bp) using the primers Comp-MAB_0545+reg-AclI/Comp-MAB_0545-HpaI and insertion into the integrative vector pMV361. The resulting construct was then introduced into the Mabs-S-Δ*lsr2* strain. Mabs-R-Δ*lsr2* complementation was achieved by PCR amplification (primer pair Comp-MAB_0545-NdeI/ Comp-MAB_0545-HindIII) and cloning of *lsr2* under the control of the *hsp60* promoter in the replicative plasmid pVV16 (Grzegorzewicz et al., 2012) Expression of *lsr2* mRNA transcripts was checked by quantitative RT-PCR analysis. To generate the Mabs-S-Δ*lsr2*-C*lsr2-FLAG* strain used for the ChIP-qPCR experiments, *lsr2* was fused to the FLAG tag by PCR amplification using the primer pair Comp-MAB_0545-reg-AclI/ Comp-MAB_0545-FLAG-HpaI and cloning with its endogenous promoter into the integrative plasmid pMVH361. All the primers used in this study are listed in Supplementary Table S1.

### Quantitative RT-PCR Analysis

mRNA was reverse transcribed using the “iScript reverse transcription” kit (Bio-Rad). Quantitative PCR was performed with a MasterMix qPCR (Eurogentec) in a Chromo4 instrument (Bio-Rad), as described previously (Pawlik et al., 2013; Le Moigne et al., 2016).

### ChIP-qPCR Analysis

For each ChIP library, 50 ml of Mabs-S-Δ*lsr2*-C*lsr2-FLAG* was grown until an OD_600_ of 0.5 and fixed in 1% formaldehyde (Euromedex) and lysed using a Precellys grinder (3 cycles: 6,700 rpm −3 × 20 s ON/60 s OFF, Bertin Technologies) and VK05 beads. The bacterial chromatin was sheared in 100–300 bp fragments using a Bioruptor Pico (Diagenode). Chromatin Immunoprecipitation was performed as described previously (Grainger et al., 2006) using IgG M2 anti-FLAG (Sigma, F1804) and Anti-GFP (G6539, Sigma) mouse monoclonal antibodies attached to proteins A/G coupled to magnetic beads (Thermo fisher). The immunoprecipitated DNA and 1% of the total input were reverse-crosslinked and eluted using the iPure v2 kit (Diagenode). The quantitative PCR tests were performed using the SsoFast evergreen supermix (Bio-Rad). Enrichment of Lsr2 binding at the GPL operon operator was calculated using the “Percent Input” method using the primers MAB_4100c_qPCR1 and MAB_4100c_qPCR3 (Supplementary Table S1).

### GPL Analysis

Apolar and polar lipid fractions were obtained from exponentially growing mycobacteria cultured as reported previously (Besra, 1998). The polar lipid fraction containing GPL was separated by TLC using silica gel 60 coated aluminum TLC plates (Merck) and chloroform/methanol (90:10; v/v) as solvent system, as previously described (Sondén et al., 2005; Pawlik et al., 2013). GPL were revealed by treating TLC plates with a mist of 0.2% (w/v) of Anthrone diluted in sulfuric acid, followed by charring.

### Susceptibility Profile to H_2_O_2_

Exponential growth phase cultures (OD_600_ between 0.6 and 0.8) of *M. abscessus* were diluted to obtain an OD_600_ of 0.1 and transferred in two new tubes. One of the two cultures was exposed to 20 mM H_2_O_2_ and the other one to an equivalent volume of sterile water. CFUs were determined by plating aliquots at various time points after the addition of H_2_O_2_ (2, 4, 6, and 8 h) on blood agar plates. For each morphotype and mutant, three independent experiments were performed, and one experiment is shown.

### Intracellular Survival in Macrophages and Amoeba After Infection

Murine J774.2 macrophages (MΦ) were grown at 37°C under 5% CO_2_ in DMEM medium supplemented with 5% heat-inactivated Fetal Calf Serum (FBS), penicillin (100 IU/ml) and streptomycin (100 μg/ml). MΦ infections with the *M. abscessus* strains were carried out, as described previously (Bakala N’Goma et al., 2015; Roux et al., 2016). CFU were counted after 3 to 5 days of incubation at 37°C. Amoeba infections were done using *Acanthamoeba castellanii* (ATCC30010), as described earlier (Bakala N’Goma et al., 2015; Dubois et al., 2018a). At 1, 2, and 3 days of co-culture, the *A. castellanii* monolayer was disrupted with 0.1% SDS for 30 min at 32°C and CFU were counted.

### Systemic Infection in Mice

Six to eight-week-old BALB/c mice were infected with *M. abscessus* strains, as described earlier (Catherinot et al., 2007; Rottman et al., 2007; Bakala N’Goma et al., 2015; Bernut et al., 2015). A Student *t*-test was carried out to test significance of differences between groups. All procedures were performed according to the institutional and national ethical guidelines and approved by the Comité d’éthique en experimentation animale N°047 with agreement A783223 under the reference APAFIS#11465.

### Zebrafish Infections

All zebrafish experiments were done according to European Union guidelines for handling of laboratory animals^1^ and approved by the Comité d’Ethique pour l’Expérimentation Animale de la région Languedoc Roussillon under the reference CEEALR36-1145. Experiments were performed using the *golden* mutant (Lamason et al., 2005). Zebrafish embryos were obtained, maintained and microinjected in the caudal vein of 30 hpf dechorionated embryos as described (Bernut et al., 2014a, 2015). Survival curves and statistics were carried out as described (Bernut et al., 2014a).

## Results

### Transcription Levels of *lsr2* in *M. abscessus* R and S Strains

**FIGURE 1:**
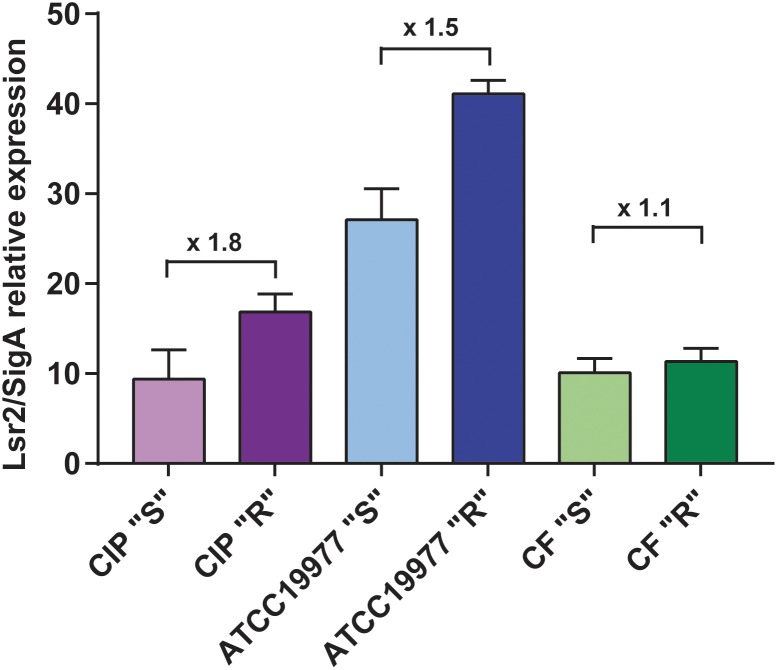
Increased transcriptional expression of *lsr2* in *M. abscessus* R variants. qRT-PCR results of *lsr2* mRNA expression in the R and S variants of *M. abscessus* CIP104536 (Collection Institut Pasteur), ATCC19977 type strains and in the CF clinical isolate. Results are expressed as *lsr2/sigA* ratio as described in Pawlik et al. (2013). For each assay, *n* = 3 and error bars are SEM.

Our initial comparative genomic analyses performed on three isogenic *M. abscessus* S/R pairs identified several mutations in the GPL biosynthesis/transport locus that are responsible for the S-to-R transition (Pawlik et al., 2013). Microarray data obtained from RNAseq highlighted the differential expression pattern of several genes, including *MAB_0545* (Pawlik et al., 2013; Supplementary Table S2). *MAB_0545*, encoding the Lsr2 protein, was found to be moderately more expressed in the R than in the S variants of the CIP104536 and ATCC19977 strains as well as in one clinical isolate, referred to as the CF R strain (Catherinot et al., 2009). To confirm these results, quantitative RT-PCR analysis was performed on *lsr2* using mRNA isolated from both the S and R variants of all three strains.Figure 1 shows that transcription of *lsr2* is increased in the R strains relative to their S counterparts, particularly in the two type strains, thus validating the microarray data. Because Lsr2 appears to be involved in the virulence and the antibiotic resistance of *M. tuberculosis* (Colangeli et al., 2009; Gordon et al., 2010), we reasoned that induction of Lsr2 expression in the R strain may explain, at least in part, the increase of virulence in the R over the S variant.

### Lsr2 Null Mutants Are Not Affected in Their Glycopeptidolipid Profile

**FIGURE 2:**
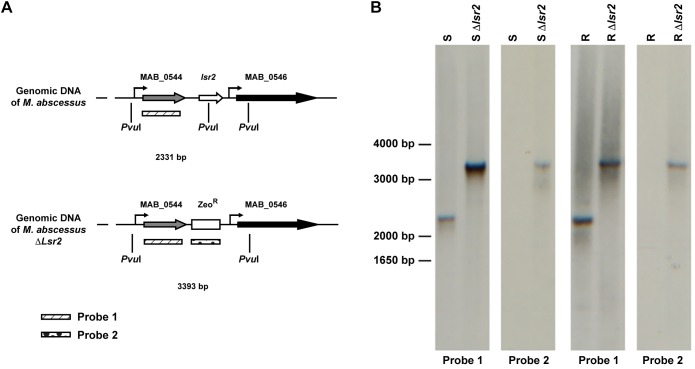
Generation of *lsr2* null mutants. **(A)** Schematic representation of the genomic regions surrounding the *lsr2* gene in the WT and *lsr2* deletion mutant. **(B)** Southern blot of Mabs-S (S), Mabs-R (R), Mabs-S-Δ*lsr2* (S Δ*lsr2*) and Mabs-R-Δ*lsr2* (R Δ*lsr2*) using Probe 1 and Probe 2 depicted in panel **(A)**. Genomic DNA was digested with the restriction enzyme *Pvu*I, migrated on an agarose gel, transferred to a nitrocellulose membrane and hybridized with the two probes. Bands of the corresponding DNA fragments observed are of the expected size.

To address the impact of Lsr2 on the *M. abscessus* morphotype, null mutants of *lsr2* were generated in both variants, designated Mabs-S-Δ*lsr2* and Mabs-R-Δ*lsr2*, using the recombineering method (van Kessel and Hatfull, 2007) that was adapted to *M. abscessus* (Bakala N’Goma et al., 2015; Halloum et al., 2016; Dubois et al., 2018b; Laencina et al., 2018). This was achieved by replacing *lsr2* with a zeocin resistance cassette using homologous recombination (Figure 2A). Proper allelic exchange was subsequently confirmed by PCR (Supplementary Figure S1) and Southern blotting (Figure 2B). qRT-PCR revealed that complementation with a functional *lsr2* gene was partial (Supplementary Figure S2).

**FIGURE 3:**
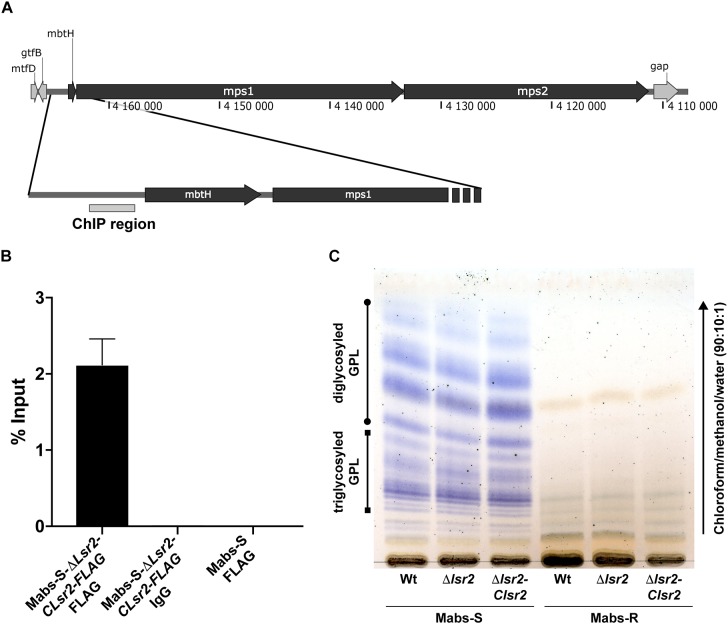
Measurement of Lsr2 enrichment in the promoter region upstream of the *mbtH/mps1* genes. **(A)** Schematic representation of the GPL locus. The gray box upstream of the *mbtH* gene represents the region used to assay Lsr2 enrichment. **(B)** qPCR of ChIP performed using an anti-FLAG antibody. Enrichment of Lsr2 bound to the *mbtH/mps1* promoter region in Mabs-S-Δ*lsr2*-*Clsr2-FLAG* was calculated using the “percent input” method (% input = 1.894%). Negative controls included in ChIP assays using an unrelated IgG antibody on Mabs-S-Δ*lsr2*-C*lsr2-FLAG* (% input = 0.00274%) and the anti-FLAG antibody on Mabs-S (% input = 0.00049%) confirm the specificity of the results. For each assay, *n* = 3 and error bars are SEM. **(C)** GPL profile characterizing the WT and Δ*lsr2* and *lsr2* complemented strains in both S and R backgrounds. Following extraction, the GPL were separated by thin layer chromatography using chloroform/methanol (90:10, v/v) and revealed with a treatment of the TLC plate with a mist of 0.2% of Anthrone in concentrated sulfuric acid and charring.

The viability of the mutant indicates that *lsr2* is dispensable in *M. abscessus*, as previously reported in *M. smegmatis* (Chen et al., 2006; Arora et al., 2008; Cortes et al., 2011). However, Mabs-S-Δ*lsr2* and Mabs-R-Δ*lsr2* colonies were heterogenous in size with a tendency toward small colonies when compared to their wild-type progenitors (Supplementary Figure S3A). All strains exhibited a similar growth rate, suggesting that the reduced colony size did not affect *in vitro* growth in this liquid medium (Supplementary Figures S3B,C). Lsr2 has been reported to be a negative regulator of GPL expression through binding to an AT-rich element upstream of the coding region of *mbtH* and *mps1*, both known to be involved in GPL production in *M. smegmatis* ATCC607 (Kocíncová et al., 2008). However, two other independent studies reported an unaltered GPL profile in *lsr2* mutants generated from the *M. smegmatis* mc^2^155 strain (Chen et al., 2006; Arora et al., 2008), which is the rough counterpart of the ATCC607 strain. We therefore addressed whether the inactivation of *lsr2* may affect the GPL profile in both S and R *M. abscessus* variants. First, chromatin immunoprecipitation was carried out on lysates of Mabs-S-Δ*lsr2* in which a *FLAG*-tagged *Lsr2* was introduced and Lsr2 enrichment in the AT-rich element within the promoter region of *mbtH/mps1* was then measured (Figure 3A). Binding of Lsr2 was found to be significantly enriched in this region (Figure 3B). However, GPL analysis by thin layer chromatography failed to show differences in the GPL profiles in Mabs-S-Δ*lsr2* and in its parental S strain (Figure 3C), suggesting that Lsr2 inactivation alone is not sufficient to alter the morphotype of the mutant. As expected, *Mabs-R*-Δ*lsr2* also failed to produce GPL, which can be explained by the presence of severe genetic lesions that impair the transcription of genes such as *mps1* and *mmpL4b* (Pawlik et al., 2013).

Collectively, these data showed Lsr2 ability to bind the promoter region upstream of *mbtH/msp1*. However, loss of Lsr2 expression has no effect on production or composition of GPL in the S *M. abscessus* variant.

### Lsr2 Promotes Resistance to Oxidative Species in the R Variant

**FIGURE 4:**
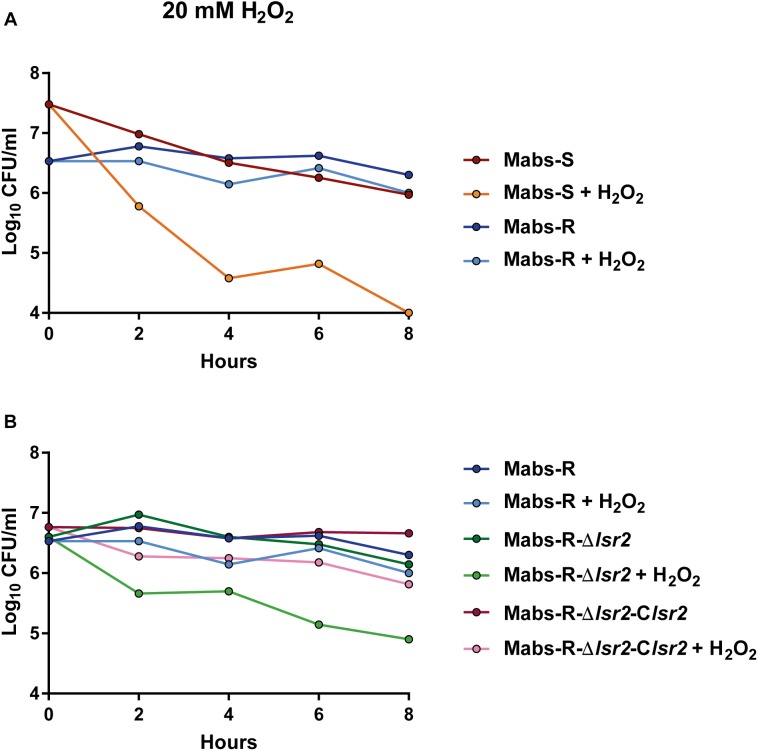
Susceptibility profile of *M. abscessus* toward oxidative derivatives. Strains were grown in broth medium until exponential phase and exposed to 20 mM H_2_O_2_. Growth was monitored by CFU counting at 2, 4, 6, and 8 h. **(A)** Comparative response of *M. abscessus* S and R variants to the treatment. **(B)** Comparative response of Mabs-R-Δ*lsr2*, Mabs-R-Δ*lsr2* and Mabs-R Δ*lsr2*-*Clsr2*.

An important function of Lsr2 resides in protecting the integrity of genomic DNA from the deleterious action of reactive oxygen species (ROS) during macrophage infection (Colangeli et al., 2009). We aimed at addressing the potential protective effect of the higher expression of *lsr2* in *M. abscessus* R cultures under H_2_O_2_ exposure. Exponential phase cultures, diluted 10 times, were exposed to 20 mM H_2_O_2_ and CFU were counted at various time points during treatment. When comparing WT *M. abscessus* S and R variants, a high proportion of the R population survived in the presence of H_2_O_2_ (*p* < 0.001) (Figure 4A). Whether this advantage is conferred by a higher *lsr2* expression was next investigated. The growth defects of Mabs-S and Mabs-S-Δ*lsr2* in the presence of H_2_O_2_ were similar (Supplementary Figure S4). In contrast, Mabs-R-Δ*lsr2* was more sensitive than its WT R progenitor, while genetic complementation in the R *lsr2* mutant restored the WT R H_2_O_2_ resistance phenotype (Figure 4B). These results show a difference in the response of the *M. abscessus* S/R variants to H_2_O_2_, the R variant being less sensitive to oxidative stress than the S variant. However, the intrinsic resistance of the R variant was partially lost upon deletion of the *lsr2* gene.

### The Intracellular Survival of *lsr2* Mutants Is Reduced in Amoebae and Macrophages

**FIGURE 5:**
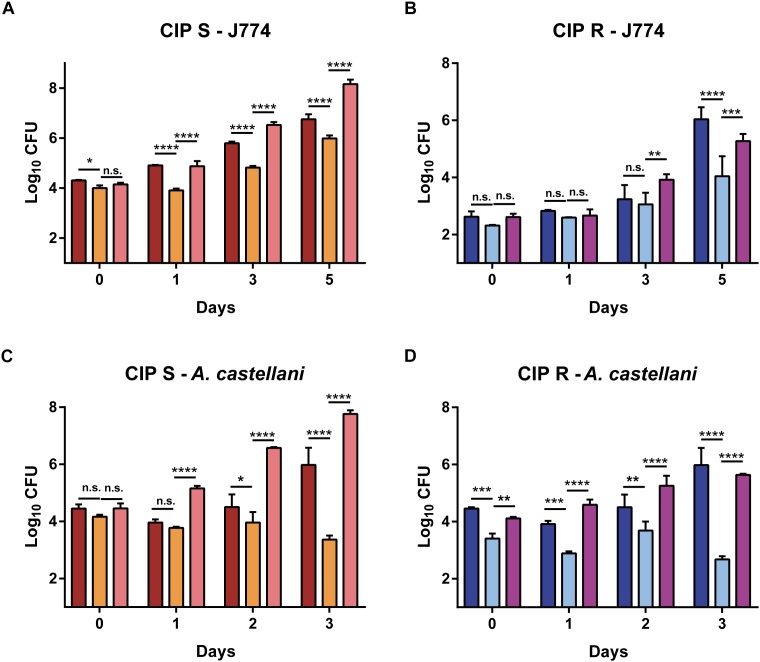
Intracellular growth of Δ*lsr2* mutants in macrophages **(A,B)** and in amoebae **(C,D)**. Murine J774.2 macrophages were infected with CIP104536 (Collection Institut Pasteur) Mabs-S (in red) and Mabs-R (dark blue), Mabs-S-Δ*lsr2* (orange) and Mabs-R-Δ*lsr2* (light blue) and their respective complemented strains (C-Δ*lsr2*; pink for S and mauve for R) at a MOI 1 whereas *A. castellanii* cells were infected with the same strains at a MOI 10. Intracellular growth was assessed by CFU counting for 5 days in macrophages and 3 days in amoebae. CFU histograms with error bars represent means ± SD calculated using data from two independent experiments. Differences between means were analyzed by two-way ANOVA and the Tukey post-test, allowing multiple comparisons. ns, non-significant, ^∗^*P* < 0.05, ^∗∗^*P* < 0.01, ^∗∗∗^*P* < 0.001, and ^∗∗∗∗^*P* < 0.0001.

The role of Lsr2 in the resistance of *M. abscessus* to oxidative species suggests that it may also protect the bacilli from the microbicidal activity of macrophages (Roux et al., 2016) and/or amoebae (Bakala N’Goma et al., 2015). This prompted us to investigate and compare the impact of *lsr2* deletion on intracellular growth of the S and R mutants with their corresponding complemented strains (Mabs-S-Δ*lsr2*-C*lsr2* and Mabs-R-Δ*lsr2*-C*lsr2*) and wild-type progenitors. Mabs-S-Δ*lsr2* (Figure 5A) and Mabs-R-Δ*lsr2* (Figure 5B) exhibited a pronounced defect inside murine MΦ as compared to the WT and complemented strains. This survival defect was already significant at 1 dpi for the S strain (*p* < 0.0001) and at 5 dpi for the R strain (*p* < 0.0001). Inside *A. castellani*, a similar decrease in bacterial viability was observed for both Mabs-S-Δ*lsr2* and Mabs-R-Δ*lsr2* mutants (Figure 5C,D). At 3 dpi, both mutants showed a strong difference (*p* < 0.0001) in intra-amoebal multiplication compared to their respective WT and complemented strains.

Together, these results indicate that Lsr2 is required for intracellular survival of *M. abscessus* S/R forms in both macrophages and amoebae.

### Lsr2 Plays a Key Role in *M. abscessus* Virulence *in vivo*

**FIGURE 6:**
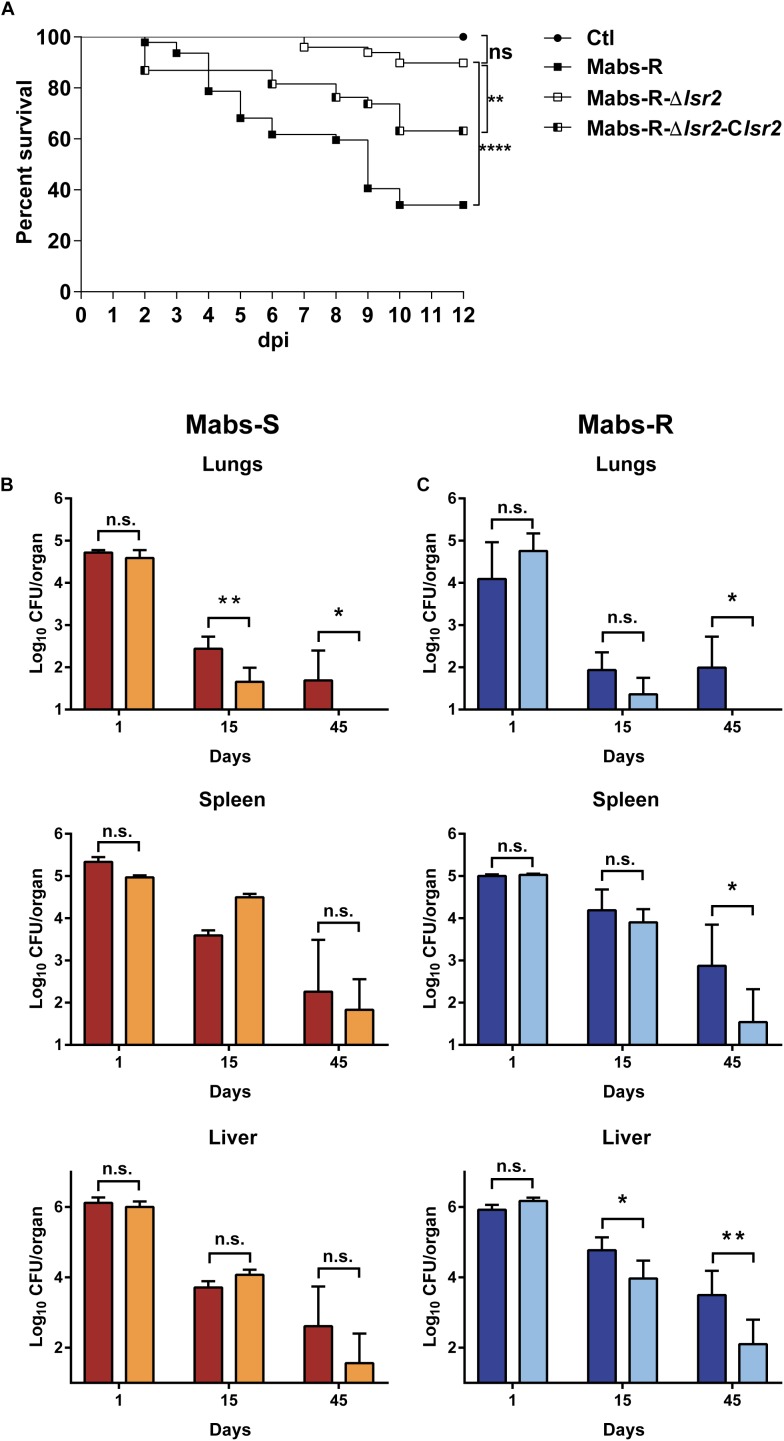
Lsr2 is required for survival of *M. abscessus* in zebrafish and mice. **(A)** Survival curve of infected embryos with 50–164 CFU of Mabs-R (*n* = 47 embryos), 160–282 CFU of Mabs-R-Δ*lsr2* (*n* = 49 embryos) and 149–290 CFU of the complemented strain (*n* = 37 embryos). Experiments were done twice. Significant differences were obtained using the Log-rank (Mantel-Cox) statistical test; ^∗∗^*P* < 0.01 and ^∗∗∗∗^*P* < 0.0001. **(B,C)** Comparative analysis of bacterial burden in different organs after autopsy of BALB/c mice infected intravenously with 10^6^ CFU of Mabs-S (in red) and Mabs-S-Δ*lsr2* (orange) **(B)** or Mabs-R (dark blue) and Mabs-R-Δ*lsr2* (light blue) **(C)** in the lungs, spleen and liver at 1, 15, and 45 dpi. Student’s *t*-test and Fisher’s exact test were used. A *P*-value of 0.05 was considered significant (^∗^*P* < 0.05 and ^∗∗^*P* < 0.01).

The impact of Lsr2 on mycobacterial virulence was originally suggested by demonstrating the preferential binding of Lsr2 to AT-rich regions in the *M. tuberculosis* genome, including several genomic islands acquired by horizontal gene transfer, encoding major virulence factors (Gordon et al., 2010). The behavior of the *lsr2* mutants was first addressed by infecting zebrafish larvae (Bernut et al., 2014a) with the wild-type R (Mabs-R) and Mabs-R-Δ*lsr2* strains. A significant reduction in the virulence of Mabs-R-Δ*lsr2* was observed when compared with Mabs-R (Figure 6A). Whereas only 10% of zebrafish were killed when infected with Mabs-R-Δ*lsr2* at 12 dpi, around 40% of the larvae died at 6 dpi with Mabs-R while nearly 70% died at 10 dpi (Figure 6A). Complementing with *lsr2* partially reversed the attenuated phenotype of Mabs-R-Δ*lsr2*.

To further evaluate the contribution of Lsr2 in pathogenesis of *M. abscessus* in a more complex animal host, BALB/c mice were intravenously infected with the wild-type S/R variants or Mabs-S/R-Δ*lsr2* strains (Catherinot et al., 2007; Rottman et al., 2007). Bacterial loads were monitored by measuring the CFU counts in the lung, spleen and liver homogenates from mice sacrificed at 1, 15, and 45 dpi. A significant reduction in the number of Mabs-S-Δ*lsr2* bacilli was observed in the lungs at 15 dpi when compared to the wild-type S progenitor (*p* < 0.01). This difference was further increased at 45 dpi, along with a complete clearance of Mabs-S-Δ*lsr2* (*p* < 0.05) (Figure 6B, upper panels). However, no significant differences in CFU counts in the spleen and the liver were observed between Mabs-S and Mabs-S-Δ*lsr2*. Monitoring the bacterial burden of Mabs-R-Δ*lsr2* revealed a significant reduction in all three organs at 45 dpi as compared to the WT parental strain (*p* < 0.05 in lungs and spleen; *p* < 0.01 in liver) (Figure 6C, lower panels).

Overall, these results indicate that Lsr2 plays a critical role in *M. abscessus* virulence in different animal hosts and highlight the requirement of Lsr2 for persistence in mice, particularly in the lungs, which represents the major targeted organ during infection in patients, especially in those with already underlying pulmonary diseases.

## Discussion

Lsr2, a unique nucleoid associated protein in the *Actinomyces* and *Mycobacterium* genera, has been extensively studied in *M. tuberculosis* (Colangeli et al., 2009; Gordon et al., 2010; Bartek et al., 2014). Like its ortholog H-NS, homodimers of Lsr2 can bind DNA cooperatively to form long oligomers on AT-rich sequences that can further interact to bridge distant DNA regions and contribute to loop formation (Chen et al., 2008; Liu and Gordon, 2012). At the genomic level, Lsr2 filaments are found at the promoters of 401 and 272 genes in *M. tuberculosis* and *M. smegmatis*, respectively, often extending into their coding regions (Gordon et al., 2010). This observation, as well as its known role as a negative regulator in several cellular functions, strongly suggest that like H-NS, Lsr2 represses transcription through promoter occlusion of RNA polymerase targets or by interfering with transcription elongation (Chen et al., 2008; Liu and Gordon, 2012; Landick et al., 2015). Lsr2 has been shown to be involved in *M. tuberculosis* virulence by binding to important genes, such as genes involved in the ESX secretion systems, the biosynthesis of the PDIM and PGL cell wall lipids or encoding antigenic proteins of the PE/PPE family (Gordon et al., 2010). Previous studies also demonstrated the implication of Lsr2 in multi-drug tolerance of *M. tuberculosis* (Colangeli et al., 2007) and in protection against reactive oxygen intermediates (Colangeli et al., 2009). All these phenotypes were investigated and confirmed in *M. abscessus*, except for the increased resistance to antibiotics (not shown), thus involving Lsr2 in increased resistance to oxidative species as well as in intracellular multiplication and survival of *M. abscessus* in zebrafish and mice.

The S-to-R transition of *M. abscessus* has only been found to occur during infection either in mice (Rottman et al., 2007) or in humans (Jönsson et al., 2007; Catherinot et al., 2009; Roux et al., 2009). In addition, the underlying genetic causes for this transition pointed to irreversible mechanisms, due to selective mutations such as indels that prevent the R form to reverse its morphotype to a S form (Pawlik et al., 2013). Thus, the S-to-R transition represents a clear advantage for *M. abscessus* during the course of the infection. When comparing pairs of isogenic S/R variants, it was found that *lsr2* was expressed at a higher level in the R morphotype (Pawlik et al., 2013). This was confirmed in the present study using qRT-PCR analysis. By inducing a higher expression of *lsr2*, the R variant becomes more resistant to reactive oxygen species. Interestingly, these conditions look similar to those prevailing in the CF airways, characterized by polymicrobial and chronic infections, which maintain a very harsh and oxidative environment in the lungs. Similarly, CF patients receive frequent cures involving large-spectrum antibiotics, such as inhaled tobramycin therapy. Herein, we show that the R form is more resistant to H_2_0_2_ than the S form. But we were unable to confirm an increased resistance to antibiotics (not shown). This resistant phenotype was lost in the *lsr2* mutant but restored to wild-type levels following complementation with a functional copy of *lsr2*.

A major outcome of this work is to point out the role of Lsr2 in the intracellular behavior of *M. abscessus* and on its requirement for persistence in infected mice and zebrafish. It was previously shown that a deletion of *lsr2* in *M. smegmatis* has an impact on its survival in murine macrophages (Colangeli et al., 2009). *In vivo* studies of the role of *lsr2* in virulence in mice were also performed on *M. tuberculosis*, using an attenuated strain that was complemented by a replicative plasmid expressing Lsr2 under the control of an inducible promoter (Ehrt et al., 2005) to circumvent the absence of viability of the *lsr2 M. tuberculosis* deletion mutant (Colangeli et al., 2007). Using this strategy, it was shown that *lsr2* is important to protect *M. tuberculosis* against ROI during macrophage infection. The present study shows that despite being dispensable in *M. abscessus*, *lsr2* remains essential for optimal intracellular growth and virulence in different vertebrates.

Previous work in *M. smegmatis* emphasized the contribution of Lsr2 in defining the lipid profile, colony morphology, bacterial motility and biofilm formation by specifically repressing transcription at the GPL locus in the rough morphotypes (Chen et al., 2006; Arora et al., 2008; Kocíncová et al., 2008; Yang et al., 2017). However, this study clearly shows that *lsr2* mRNAs are detected in both *M. abscessus* S and R strains. Thanks to ChIP-qPCR, we also observed that Lsr2 is able to bind the promoter region of *mbtH* in *M. abscessus* S, despite the equivalent GPL profiles in the WT and the mutant strain. This suggests that binding of Lsr2 to this particular genomic region is not sufficient to regulate the biosynthetic *GPL* locus and that this process may involve other partners that remain to be identified. An alternative explanation is that the higher expression level of Lsr2 in *M. abscessus* R is needed to successfully reduce GPL production, eventually by increasing the formation of a DNA loop that can occlude RNA polymerase binding or inhibit transcription elongation, as proposed for H-NS in Gram-negative bacteria (Landick et al., 2015). One would have anticipated that increasing expression of Lsr2 in *M. abscessus* R would enhance the R phenotype by further repressing the genes of the GPL locus. However, deletion of *lsr2* in *M. abscessus* R did not restore GPL production as it was previously observed in *M. smegmatis* (Kocíncová et al., 2008). As mentioned above, the GPL locus in the *M. abscessus* R variant suffers from irreversible genetic lesions, adding a layer of complexity to the molecular mechanisms involved in the regulation of this locus. The pleiotropic nature of Lsr2 implies that its deletion has a broader impact on the fitness of *M. abscessus* as well as on the general compaction of its nucleoid. Thus, additional analysis using genome-wide methods will help to unravel the details of the molecular role of Lsr2 during S-to-R transition and its impact on pathogenicity.

Taken together, this work showed the impact of Lsr2 as a key virulence factor on the intracellular and *in vivo* survival of *M. abscessus*. These results also strongly suggest that Lsr2, like other NAPs, might represent a target of choice for the development of new antimicrobials. Indeed, compounds that would either silence expression of lsr2 or inhibit its function could therefore be considered as a new anti-virulence approach against *M. abscessus* (Liu and Gordon, 2012).

## Ethics Statement

All procedures involving mice were performed according to the institutional and national ethical guidelines and approved by the comité d’éthique en experimentation animale N°047 with agreement A783223 under the reference APAFIS#11465. All zebrafish experiments were done according to European Union guidelines for handling of laboratory animals (http://ec.europa.eu/environment/chemicals/lab_animals/home_en.htm) and approved by the Comité d’Ethique pour l’Expérimentation Animale de la région Languedoc Roussillon under the reference CEEALR36-1145.

## Author Contributions

J-LH, LK, and FC designed the project and experiments. VLM, AB, MC, AV, CD, and AP performed the experiments. J-LG, FM, FC, LK, and J-LH wrote and corrected the manuscript.

## Conflict of Interest Statement

The authors declare that the research was conducted in the absence of any commercial or financial relationships that could be construed as a potential conflict of interest.

## Footnotes

1 http://ec.europa.eu/environment/chemicals/lab_animals/home_en.htm

## References

[B1] Arora K. Whiteford D. C. Lau-Bonilla D. Davitt C. M. Dahl J. L. (2008). Inactivation of *lsr2* results in a hypermotile phenotype in *Mycobacterium smegmatis*. *J. Bacteriol.* 190 4291–4300. 10.1128/JB.00023-08 18408023 PMC2446759

[B2] Bakala N’Goma J. C. Le Moigne V. Soismier N. Laencina L. Le Chevalier F. (2015). *Mycobacterium abscessus* phospholipase C expression is induced during coculture within amoebae and enhances *M. abscessus* virulence in mice. *Infect. Immun.* 83 780–791. 10.1128/IAI.02032-14 25486995 PMC4294255

[B3] Bartek I. L. Woolhiser L. K. Baughn A. D. Basaraba R. J. Jacobs W. R. Jr. Lenaerts A. J. (2014). *Mycobacterium tuberculosis* Lsr2 is a global transcriptional regulator required for adaptation to changing oxygen levels and virulence. *mBio* 5:e01106-14. 10.1128/mBio.01106-14 24895305 PMC4049101

[B4] Bernut A. Dupont C. Sahuquet A. Herrmann J.-L. Lutfalla G. Kremer L. (2015). Deciphering and Imaging Pathogenesis and Cording of *Mycobacterium abscessus* in zebrafish embryos. *J. Vis. Exp.* 103:53130. 10.3791/53130 26382225 PMC4692601

[B5] Bernut A. Herrmann J.-L. Kissa K. Dubremetz J.-F. Gaillard J.-L. Lutfalla G. (2014a). *Mycobacterium abscessus* cording prevents phagocytosis and promotes abscess formation. *Proc. Natl. Acad. Sci. U.S.A.* 111 E943–E952. 10.1073/pnas.1321390111 24567393 PMC3956181

[B6] Bernut A. Le Moigne V. Lesne T. Lutfalla G. Herrmann J.-L. Kremer L. (2014b). In vivo assessment of drug efficacy against *Mycobacterium abscessus* using the embryonic zebrafish test system. *Antimicrob. Agents Chemother.* 58 4054–4063. 10.1128/AAC.00142-14 24798271 PMC4068527

[B7] Bernut A. Herrmann J.-L. Ordway D. Kremer L. (2017). The diverse cellular and animal models to decipher the physiopathological traits of *Mycobacterium abscessus* infection. *Front. Cell. Infect. Microbiol.* 7:100. 10.3389/fcimb.2017.00100 28421165 PMC5378707

[B8] Besra G. S. (1998). Preparation of cell-wall fractions from mycobacteria. *Methods Mol. Biol.* 101 91–107. 9921472 10.1385/0-89603-471-2:91

[B9] Brown-Elliott B. A. Wallace R. J. (2002). Clinical and taxonomic status of pathogenic nonpigmented or late-pigmenting rapidly growing mycobacteria. *Clin. Microbiol. Rev.* 15 716–746. 12364376 10.1128/CMR.15.4.716-746.2002 PMC126856

[B10] Byrd T. F. Lyons C. R. (1999). Preliminary characterization of a *Mycobacterium abscessus* mutant in human and murine models of infection. *Infect. Immun.* 67 4700–4707. 10456919 10.1128/iai.67.9.4700-4707.1999 PMC96797

[B11] Catherinot E. Clarissou J. Etienne G. Ripoll F. Emile J.-F. Daffé M. (2007). Hypervirulence of a rough variant of the *Mycobacterium abscessus* type strain. *Infect. Immun.* 75 1055–1058. 17145951 10.1128/IAI.00835-06 PMC1828507

[B12] Catherinot E. Roux A.-L. Macheras E. Hubert D. Matmar M. Dannhoffer L. (2009). Acute respiratory failure involving an R variant of *Mycobacterium abscessus*. *J. Clin. Microbiol.* 47 271–274. 10.1128/JCM.01478-08 19020061 PMC2620830

[B13] Chen J. M. German G. J. Alexander D. C. Ren H. Tan T. Liu J. (2006). Roles of Lsr2 in colony morphology and biofilm formation of *Mycobacterium smegmatis*. *J. Bacteriol.* 188 633–641. 16385053 10.1128/JB.188.2.633-641.2006 PMC1347275

[B14] Chen J. M. Ren H. Shaw J. E. Wang Y. J. Li M. Leung A. S. (2008). Lsr2 of *Mycobacterium tuberculosis* is a DNA-bridging protein. *Nucleic Acids Res.* 36 2123–2135. 10.1093/nar/gkm1162 18187505 PMC2367712

[B15] Colangeli R. Haq A. Arcus V. L. Summers E. Magliozzo R. S. McBride A. (2009). The multifunctional histone-like protein Lsr2 protects mycobacteria against reactive oxygen intermediates. *Proc. Natl. Acad. Sci. U.S.A.* 106 4414–4418. 10.1073/pnas.0810126106 19237572 PMC2657463

[B16] Colangeli R. Helb D. Vilchèze C. Hazbon M. H. Lee C.-G. Safi H. (2007). Transcriptional regulation of multi-drug tolerance and antibiotic-induced responses by the histone-like protein Lsr2 in *M. tuberculosis*. *PLoS Pathog.* 3:e87. 10.1371/journal.ppat.0030087 17590082 PMC1894825

[B17] Cortes M. Singh A. K. Reyrat J. M. Gaillard J.-L. Nassif X. Herrmann J.-L. (2011). Conditional gene expression in *Mycobacterium abscessus*. *PLoS One* 6:e29306. 10.1371/journal.pone.0029306 22195042 PMC3240655

[B18] Diel R. Ringshausen F. Richter E. Welker L. Schmitz J. Nienhaus A. (2017). Microbiological and clinical outcomes of treating non-*Mycobacterium avium* complex nontuberculous mycobacterial pulmonary disease: a systematic review and meta-analysis. *Chest* 152 120–142. 10.1016/j.chest.2017.04.166 28461147

[B19] Dubois V. Laencina L. Bories A. Le Moigne V. Pawlik A. Herrmann J. L. (2018a). Identification of virulence markers of *Mycobacterium abscessus* for intracellular replication in phagocytes. *J. Vis. Exp.* 139:e57766. 10.3791/57766 30320743 PMC6235313

[B20] Dubois V. Viljoen A. Laencina L. Le Moigne V. Bernut A. Dubar F. (2018b). MmpL8MAB controls *Mycobacterium abscessus* virulence and production of a previously unknown glycolipid family. *Proc. Natl. Acad. Sci. U.S.A.* 115 E10147–E10156. 10.1073/pnas.1812984115 30301802 PMC6205491

[B21] Ehrt S. Guo X. V. Hickey C. M. Ryou M. Monteleone M. Riley L. W. (2005). Controlling gene expression in mycobacteria with anhydrotetracycline and Tet repressor. *Nucleic Acids Res.* 33:e21. 10.1093/nar/gni013 15687379 PMC548372

[B22] Esther C. R. Esserman D. A. Gilligan P. Kerr A. Noone P. G. (2010). Chronic *Mycobacterium abscessus* infection and lung function decline in cystic fibrosis. *J. Cyst. Fibros. Soc.* 9 117–123. 10.1016/j.jcf.2009.12.001 20071249 PMC3837580

[B23] Gordon B. R. Imperial R. Wang L. Navarre W. W. Liu J. (2008). Lsr2 of *Mycobacterium* represents a novel class of H-NS like proteins. *J. Bacteriol.* 190 7052–7059. 10.1128/JB.00733-08 18776007 PMC2580683

[B24] Gordon B. R. Li Y. Wang L. Sintsova A. van Bakel H. Tian S. (2010). Lsr2 is a nucleoid-associated protein that targets AT-rich sequences and virulence genes in *Mycobacterium tuberculosis*. *Proc. Natl. Acad. Sci. U.S.A.* 107 5154–5159. 10.1073/pnas.0913551107 20133735 PMC2841939

[B25] Gordon B. R. G. Li Y. Cote A. Weirauch M. T. Ding P. Hughes T. R. (2011). Structural basis for recognition of AT-rich DNA by unrelated xenogeneic silencing proteins. *Proc. Natl. Acad. Sci. U.S.A.* 108 10690–10695. 10.1073/pnas.1102544108 21673140 PMC3127928

[B26] Grainger D. C. Hurd D. Goldberg M. D. Busby S. J. (2006). Association of nucleoid proteins with coding and non-coding segments of the *Escherichia coli* genome. *Nucleic Acids Res.* 34 4642–4652. 10.1093/nar/gkl542 16963779 PMC1636352

[B27] Grzegorzewicz A. E. Pham H. Gundi V. A. K. B. Scherman M. S. North E. J. Hess T. (2012). Inhibition of mycolic acid transport across the *Mycobacterium tuberculosis* plasma membrane. *Nat. Chem. Biol.* 8 334–341. 10.1038/nchembio.794 22344175 PMC3307863

[B28] Gutiérrez A. V. Viljoen A. Ghigo E. Herrmann J.-L. Kremer L. (2018). Glycopeptidolipids, a double-edged sword of the *Mycobacterium abscessus* complex. *Front. Microbiol.* 9:1145. 10.3389/fmicb.2018.01145 29922253 PMC5996870

[B29] Halloum I. Carrère-Kremer S. Blaise M. Viljoen A. Bernut A. Le Moigne V. (2016). Deletion of a dehydratase important for intracellular growth and cording renders rough *Mycobacterium abscessus* avirulent. *Proc. Natl. Acad. Sci. U.S.A.* 113 E4228–E4237. 10.1073/pnas.1605477113 27385830 PMC4961194

[B30] Howard S. T. Rhoades E. Recht J. Pang X. Alsup A. Kolter R. (2006). Spontaneous reversion of *Mycobacterium abscessus* from a smooth to a rough morphotype is associated with reduced expression of glycopeptidolipid and reacquisition of an invasive phenotype. *Microbiology* 152 1581–1590. 16735722 10.1099/mic.0.28625-0

[B31] Jeong S. H. Kim S.-Y. Huh H. J. Ki C.-S. Lee N. Y. Kang C.-I. (2017). Mycobacteriological characteristics and treatment outcomes in extrapulmonary *Mycobacterium abscessus* complex infections. *Int. J. Infect. Dis.* 60 49–56. 10.1016/j.ijid.2017.05.007 28522316

[B32] Jönsson B. E. Gilljam M. Lindblad A. Ridell M. Wold A. E. Welinder-Olsson C. (2007). Molecular epidemiology of *Mycobacterium abscessus*, with focus on cystic fibrosis. *J. Clin. Microbiol.* 45 497–504. 17376883 10.1128/JCM.02592-06 PMC1865885

[B33] Kocíncová D. Singh A. K. Beretti J. L. Ren H. Euphrasie D. Liu J. (2008). Spontaneous transposition of IS1096 or ISMsm3 leads to glycopeptidolipid overproduction and affects surface properties in *Mycobacterium smegmatis*. *Tuberculosis* 88 390–398. 10.1016/j.tube.2008.02.005 18439873

[B34] Laal S. Sharma Y. D. Prasad H. K. Murtaza A. Singh S. Tangri S. (1991). Recombinant fusion protein identified by lepromatous sera mimics native *Mycobacterium leprae* in T-cell responses across the leprosy spectrum. *Proc. Natl. Acad. Sci. U.S.A.* 88 1054–1058. 1992456 10.1073/pnas.88.3.1054 PMC50953

[B35] Laencina L. Dubois V. Le Moigne V. Viljoen A. Majlessi L. Pritchard J. (2018). Identification of genes required for *Mycobacterium abscessus* growth in vivo with a prominent role of the ESX-4 locus. *Proc. Natl. Acad. Sci. U.S.A.* 115 E1002–E1011. 10.1073/pnas.1713195115 29343644 PMC5798338

[B36] Lamason R. L. Mohideen M. A. Mest J. R. Wong A. C. Norton H. L. Aros M. C. (2005). SLC24A5, a putative cation exchanger, affects pigmentation in zebrafish and humans. *Science* 310 1782–1786. 16357253 10.1126/science.1116238

[B37] Landick R. Wade J. T. Grainger D. C. (2015). H-NS and RNA polymerase: a love–hate relationship? *Curr. Opin. Microbiol.* 24 53–59. 10.1016/j.mib.2015.01.009 25638302

[B38] Le Moigne V. Belon C. Goulard C. Accard G. Bernut A. Pitard B. (2016). MgtC as a host-induced factor and vaccine candidate against *Mycobacterium abscessus* infection. *Infect. Immun.* 84 2895–2903. 10.1128/IAI.00359-16 27481243 PMC5038086

[B39] Liu J. Gordon B. R. (2012). Targeting the global regulator Lsr2 as a novel approach for anti-tuberculosis drug development. *Expert Rev. Anti. Infect. Ther.* 10 1049–1053. 10.1586/eri.12.86 23106279

[B40] Medjahed H. Gaillard J.-L. Reyrat J.-M. (2010). *Mycobacterium abscessus*: a new player in the mycobacterial field. *Trends Microbiol.* 18 17–23. 10.1016/j.tim.2009.12.007 20060723

[B41] Medjahed H. Reyrat J.-M. (2009). Construction of *Mycobacterium abscessus* defined glycopeptidolipid mutants: comparison of genetic tools. *Appl. Environ. Microbiol.* 75 1331–1338. 10.1128/AEM.01914-08 19114521 PMC2648176

[B42] Mougari F. Guglielmetti L. Raskine L. Sermet-Gaudelus I. Veziris N. Cambau E. (2016). Infections caused by *Mycobacterium abscessus*: epidemiology,diagnostic tools and treatment. *Expert Rev. Anti. Infect. Ther.* 14 1139–1154. 27690688 10.1080/14787210.2016.1238304

[B43] Nessar R. Reyrat J.-M. Davidson L. B. Byrd T. F. (2011). Deletion of the mmpL4b gene in the *Mycobacterium abscessus* glycopeptidolipid biosynthetic pathway results in loss of surface colonization capability, but enhanced ability to replicate in human macrophages and stimulate their innate immune response. *Microbiology* 157 1187–1195. 10.1099/mic.0.046557-0 21292749

[B44] Nguyen K. T. Piastro K. Gray T. A. Derbyshire K. M. (2010). Mycobacterial biofilms facilitate horizontal DNA transfer between strains of *Mycobacterium smegmatis*. *J. Bacteriol.* 192 5134–5142. 10.1128/JB.00650-10 20675473 PMC2944546

[B45] Oberley-Deegan R. E. Rebits B. W. Weaver M. R. Tollefson A. K. Bai X. McGibney M. (2010). An oxidative environment promotes growth of *Mycobacterium abscessus*. *Free Radic. Biol. Med.* 49 1666–1673. 10.1016/j.freeradbiomed.2010.08.026 20807564 PMC2970643

[B46] Pawlik A. Garnier G. Orgeur M. Tong P. Lohan A. Le Chevalier F. (2013). Identification and characterization of the genetic changes responsible for the characteristic smooth-to-rough morphotype alterations of clinically persistent *Mycobacterium abscessus*. *Mol. Microbiol.* 90 612–629. 10.1111/mmi.12387 23998761

[B47] Qu Y. Lim C. J. Whang Y. R. Liu J. Yan J. (2013). Mechanism of DNA organization by *Mycobacterium tuberculosis* protein Lsr2. *Nucleic Acids Res.* 41 5263–5272. 10.1093/nar/gkt249 23580555 PMC3664827

[B48] Qvist T. Taylor-Robinson D. Waldmann E. Olesen H. V. Hansen C. R. Mathiesen I. H. (2016). Comparing the harmful effects of nontuberculous mycobacteria and Gram negative bacteria on lung function in patients with cystic fibrosis. *J. Cyst. Fibros.* 15 380–385. 10.1016/j.jcf.2015.09.007 26482717 PMC4893021

[B49] Ripoll F. Pasek S. Schenowitz C. Dossat C. Barbe V. Rottman M. (2009). Non-mycobacterial virulence genes in the genome of the emerging pathogen *Mycobacterium abscessus*. *PLoS One* 4:e5660. 10.1371/journal.pone.0005660 19543527 PMC2694998

[B50] Rottman M. Soudais C. Vogt G. Renia L. Emile J.-F. Decaluwe H. (2007). Importance of T cells, gamma interferon, and tumor necrosis factor in immune control of the rapid grower *Mycobacterium abscessus* in C57BL/6 mice. *Infect. Immun.* 75 5898–5907. 17875636 10.1128/IAI.00014-07 PMC2168332

[B51] Roux A.-L. Catherinot E. Ripoll F. Soismier N. Macheras E. Ravilly S. (2009). Multicenter study of prevalence of nontuberculous mycobacteria in patients with cystic fibrosis in France. *J. Clin. Microbiol.* 47 4124–4128. 19846643 10.1128/JCM.01257-09 PMC2786646

[B52] Roux A.-L. Ray A. Pawlik A. Medjahed H. Etienne G. Rottman M. (2011). Overexpression of proinflammatory TLR-2-signalling lipoproteins in hypervirulent mycobacterial variants. *Cell. Microbiol.* 13 692–704. 10.1111/j.1462-5822.2010.01565.x 21143571

[B53] Roux A.-L. Viljoen A. Bah A. Simeone R. Bernut A. Laencina L. (2016). The distinct fate of smooth and rough *Mycobacterium abscessus* variants inside macrophages. *Open Biol.* 6:160185. 27906132 10.1098/rsob.160185 PMC5133439

[B54] Sánchez-Chardi A. Olivares F. Byrd T. F. Julián E. Brambilla C. Luquin M. (2011). Demonstration of cord formation by rough *Mycobacterium abscessus* variants: implications for the clinical microbiology laboratory. *J. Clin. Microbiol.* 49 2293–2295. 10.1128/JCM.02322-10 21490192 PMC3122772

[B55] Smibert O. Snell G. I. Bills H. Westall G. P. Morrissey C. O. (2016). *Mycobacterium abscessus* complex - a particular challenge in the setting of lung transplantation. *Expert Rev. Anti. Infect. Ther.* 14 325–333. 26732819 10.1586/14787210.2016.1138856

[B56] Sondén B. Kocíncová D. Deshayes C. Euphrasie D. Rhayat L. Laval F. (2005). Gap, a mycobacterial specific integral membrane protein, is required for glycolipid transport to the cell surface. *Mol. Microbiol.* 58 426–440. 16194230 10.1111/j.1365-2958.2005.04847.x

[B57] van Dorn A. (2017). Multidrug-resistant *Mycobacterium abscessus* threatens patients with cystic fibrosis. *Lancet Respir. Med.* 5:15. 27956215 10.1016/S2213-2600(16)30444-1

[B58] van Kessel J. C. Hatfull G. F. (2007). Recombineering in *Mycobacterium tuberculosis*. *Nat. Methods* 4 147–152. 17179933 10.1038/nmeth996

[B59] Varghese B. Shajan S. E. Al Saedi M. O. Al-Hajoj S. A. (2012). First case report of chronic pulmonary lung disease caused by *Mycobacterium abscessus* in two immunocompetent patients in Saudi Arabia. *Ann. Saudi Med.* 32 312–314. 10.5144/0256-4947.2012.312 22588446 PMC6081043

[B60] Yang Y. Thomas J. Li Y. Vilchèze C. Derbyshire K. M. Jacobs W. R. (2017). Defining a temporal order of genetic requirements for development of mycobacterial biofilms. *Mol. Microbiol.* 105 794–809. 10.1111/mmi.13734 28628249 PMC5607029

