# Supplementary material for: Lsr2 Is an Important Determinant of Intracellular Growth and Virulence in Mycobacterium abscessus
Source: Front Microbiol. 2019 Apr 30;10:905. doi: 10.3389/fmicb.2019.00905 (PMC6503116; doi:10.3389/fmicb.2019.00905)
Supplement: Supplementary file 1 [file Data_Sheet_1.PDF]

Supplementary data:

Table S1: Primers used in this study.

Name	Sequence
MC75	5’-cgaagcttgacttcttggagttggagac-3’
MC76	5’-cgcatatgtagatcagagtgtgggtcgc-3’
MC80	5’-gcggatccgttcccgggacaatttcg-3’
MC81	5’-cgtctagacccgctcgatttccttc-3’
lsr2-5	5’-gcaatgcatgctaagaaggtcacggtcacg-3’
lsr2V	5’-acattgtgcccgttcttac-3’
ZeoR	5’-cgactagtgatccccgggaattc-3’
Comp-MAB_0545+reg-AclI	5’-ggaattcaacgttcttttcctgaaaaaggcactcg-3’
Comp-MAB_0545-HpaI	5’-ggaattcgttaacctagttggccgcgttgaac-3’
Comp-MAB_0545-NdeI	5’-ggaattccatatgatggctaagaaggtcacggt-3’
Comp-MAB_0545-HindIII	5’-atatccaagcttctagttggccgcgttgaac-3’
Comp-MAB_0545-FLAG-HpaI	5’gaattcgttaaccta**cttgtcgtcgtcgtccttgtagtcgatgtcg tggtccttgtagtcaccgtcgtggtccttgtagtc**gttggccgcgtt gaacgc-3’
MAB_4100c_qPCR1	5’-gtggacatcgcaaacatacg-3’
MAB_4100c_qPCR3	5’-cgaatgggaatgtgtcaaag-3’
Lsr2-QPCR-F	5’-gagaccgttgaattcggtg-3’
Lsr2-QPCR-R	5’-gctgattacgcagcttctcc-3’
SigA1F	5’-tccgagaaagacaaggcttc-3’
SigA1R’	5’-ccagctcgacttcctcttcg-3’

Table S2. ***lsr2*** **mRNA ratio between R and S variants of** ***M. abscessus*** **CIP104536 (Collection Institut Pasteur), ATCC19977 type strains and from the CF clinical isolate, from Pawlik** ***et al*****. (2013).**

**Compared strains**	**19977-IP-R/ 19977-IP-S**	**19977-AT-R/ 19977-AT-S**	**CF-R/ CF-S**
**Ratio (Standard deviation)**	**1.311 (+/- 0.007)**	**1.654 (+/- 0.011)**	**1.250 (+/- 0.003)**

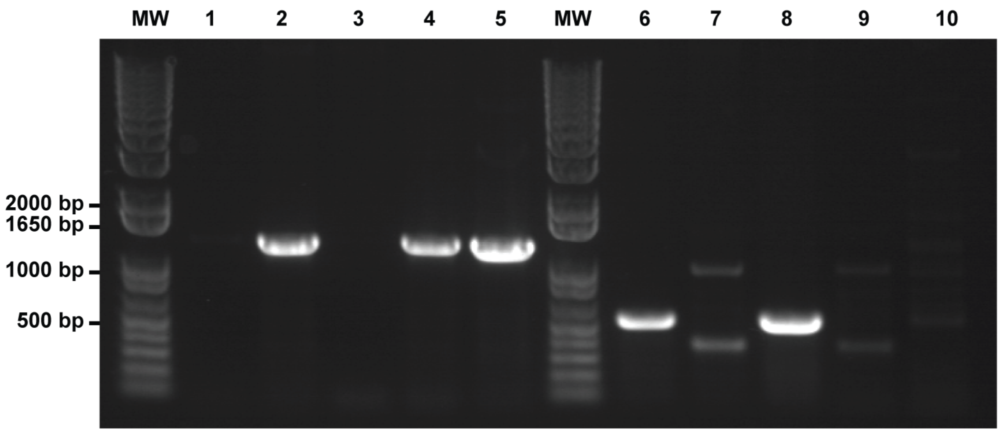

Figure S1. **PCR validation of *lsr2* knock-out mutants (Δ*lsr2*).** Columns 1 to 5: PCR carried out with primers MC75 and ZeoR (no amplification in Mabs-S or -R wild-type strains, 1356 bp fragment for Mabs-S and -R-Δ*lsr2*). Columns 6 to 10: PCR carried out with the primers lsr2-5 and lsr2V (fragment of 552bp for Mabs-S or -R wild-type strains and no amplification for Mabs-S and -R-Δ*lsr2*). Columns 1 and 6: Mabs-S; columns 2 and 7: Mabs-S-Δ*lsr2*; columns 3 and 8: Mabs-R; columns 4 and 9: Mabs-R-Δ*lsr2*; columns 5 and 10: pMC34 (control plasmid containing the AES); MW columns: Molecular weight marker. The bands correspond to the expected results.

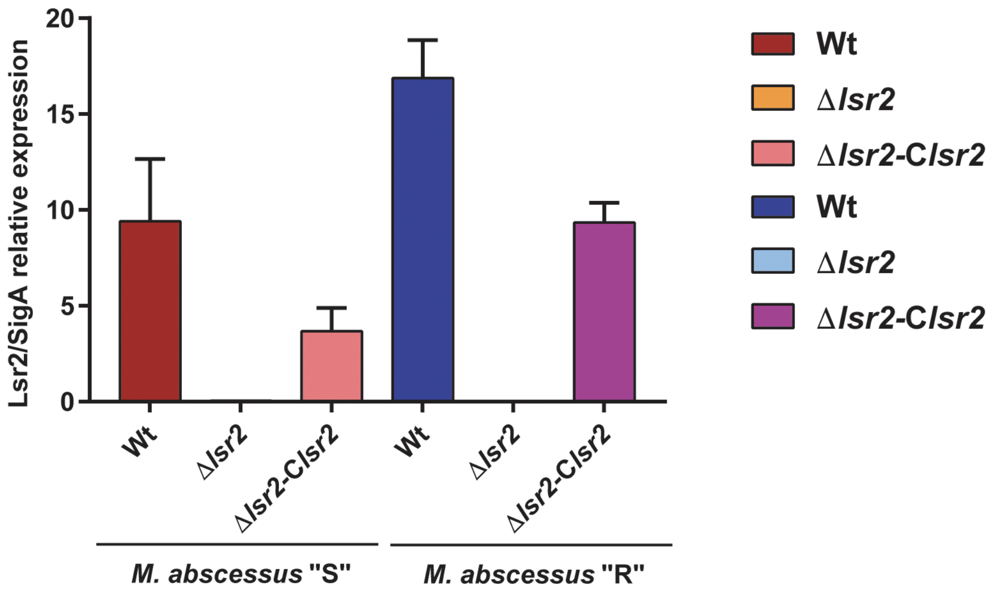

Figure S2. Absence of *lsr2* expression in the *lsr2* knock-out strains, while transcripts of the gene are detected in the complemented strains using RT-qPCR.

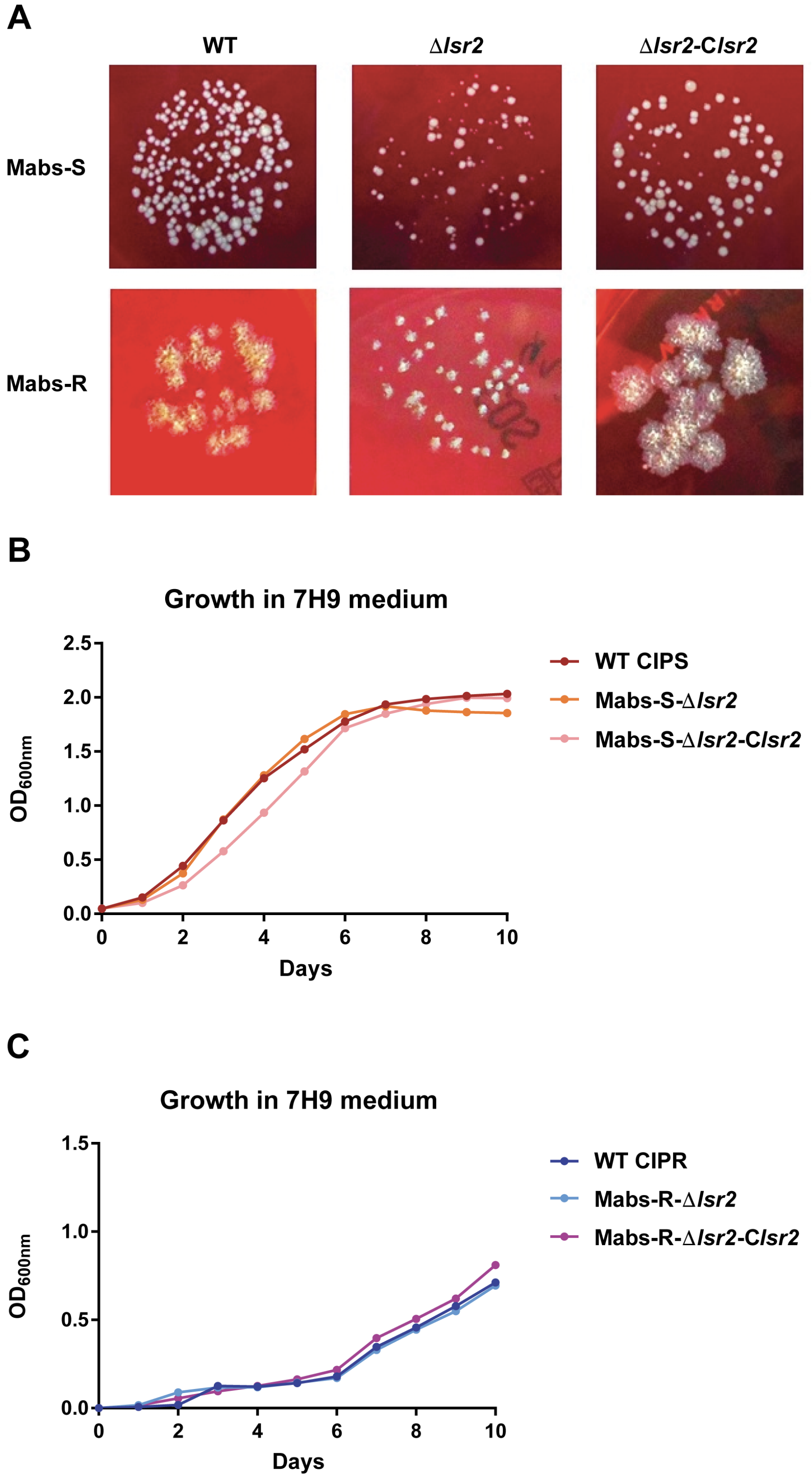

Figure S3. (A) Photographs of the colonies from the S and R WT type 19977-IP and their corresponding mutant and complemented strains. **(B)** Growth curve showing the absence of growth defect in Mabs-S-Δ*lsr2* as compared to Mabs-S and Mabs-S-Δ*lsr2*-C*lsr2* strains. **(C)** Growth curve showing the absence of growth defect in Mabs-R-Δ*lsr2* as compared to Mabs-R and Mabs-R-Δ*lsr2*-C*lsr2* strains.

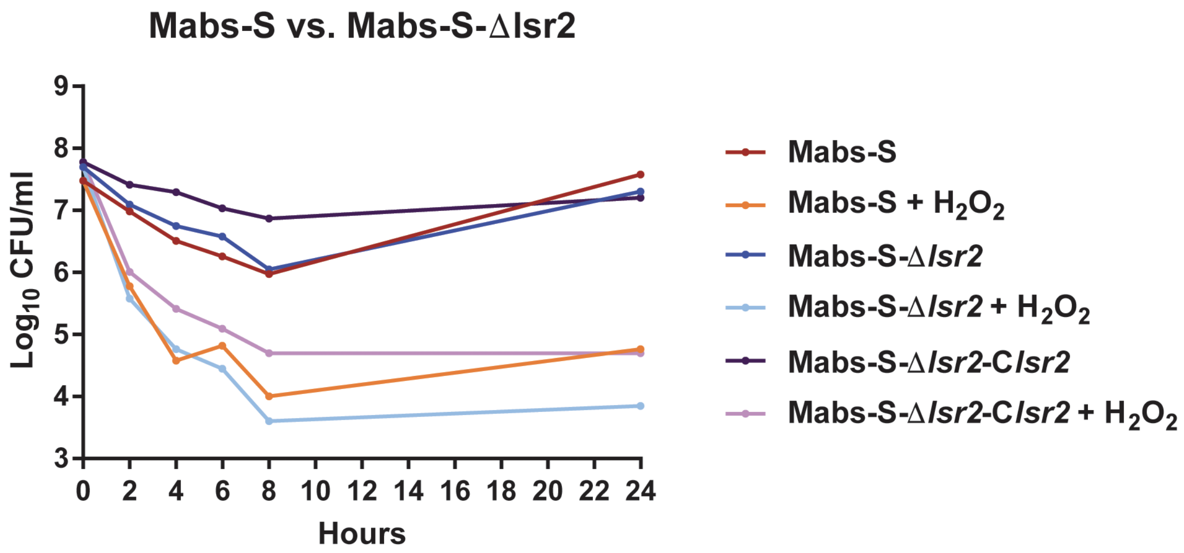

Figure S4. Resistance to H_2O_2 of Mabs-S versus Mabs-S-Δ*lsr2* and Mabs-S-Δ*lsr2*-Clsr2. As described in material and methods, Mabs-S, Mabs-S-Δ*lsr2* and Mabs-S-Δ*lsr2*-Clsr2 were grown in liquid medium up to exponential phase of growth. H_2O_2 was then added at 20 mM concentration and decrease of growth was monitored by CFU counting at 2, 4, 6, 8, and 24 h.
